# Insulin-like growth factor 1 associated research in Alzheimer’s disease: an exploratory trends analysis

**DOI:** 10.3389/fneur.2026.1709559

**Published:** 2026-03-05

**Authors:** Yan-Jun Chen, Ming-Rong Xie, Qin-Quan Zhu, Sheng-Qiang Zhou, Bo Li, Hui Yuan

**Affiliations:** 1 Graduate School of Hunan University of Chinese Medicine, Changsha, China; 2 The First Clinical College of Nanjing University of Chinese Medicine, Nanjing, China; 3 The First Hospital of Hunan University of Chinese Medicine, Changsha, China; 4 National TCM Master Liu Zuyi Inheritance Studio, Hunan Provincial Hospital of Integrated Traditional Chinese and Western Medicine (The Affiliated Hospital of Hunan Academy of Traditional Chinese Medicine), Changsha, China; 5 Department of Chinese Medicine, Hunan Aerospace Hospital, The Affiliated Aerospace Hospital of Hunan Normal University, Changsha, China

**Keywords:** Alzheimer’s disease, cognitive impairment, depression, IGF-1, metabolism, oxidative stress

## Abstract

**Background:**

Alzheimer’s disease (AD) is a neurodegenerative disorder characterized by progressive cognitive impairment and behavioral deterioration. In recent years, the role of the insulin-like growth factor-1 (IGF-1) signaling pathway in the pathological process of AD has received increasing attention. This study provides a visual analysis of the current research status, development trends, collaboration networks, and research hotspots related to IGF-1 and AD.

**Methods:**

Publications were retrieved from the Web of Science and Scopus databases. CiteSpace, VOSviewer, and Bibliometrix software were used for visual analysis.

**Results:**

A total of 632 publications were included in the study. The annual publications related to IGF-1 and AD exhibited an overall upward trend. Research was concentrated in North America, Asia, and Europe. The United States holds a dominant position in terms of output, influence, and international influence. The Consejo Superior de Investigaciones Científicas was the most active institution. *Journal of Alzheimer’s Disease* was the journal with the highest number of publications. Dr. Ignacio Torres-Aleman was the most prolific author. High-frequency keywords included IGF-1, AD, brain, insulin, controlled study, metabolism, oxidative stress, animals, signal transduction, amyloid beta protein, dementia, aging, and neuroprotection. Transgenic mouse, risk, depression, and cognitive impairment were the most powerful keywords that have emerged in recent years.

**Conclusion:**

Research on IGF-1 and AD has continued to grow. Studies in this field have formed a tightly interconnected network, centered on the AD pathological core—IGF-1-related molecular mechanisms—downstream signaling pathways. The research focus is shifting from superficial correlations to investigations into underlying mechanisms and potential therapeutic targets. Depression and cognitive impairment are likely to become promising frontiers for future research.

## 1 Introduction

Alzheimer’s disease (AD) is a neurodegenerative disorder of the central nervous system characterized by progressive cognitive impairment and behavioral deterioration (1). The pathogenesis of AD is complex, and the mainstream theory holds that its core pathological features are the extracellular deposition of β-amyloid protein (Aβ), forming senile plaques, and the intracellular accumulation of excessive phosphorylated Tau protein, creating neurofibrillary tangles (2). The imbalance in the generation and clearance of Aβ triggers the “amyloid cascade hypothesis,” which subsequently leads to Tau pathology, synaptic dysfunction, neuroinflammation, oxidative stress, and ultimately neuronal loss (3, 4). The main treatments for AD currently include cholinesterase inhibitors, NMDA receptor antagonists, and monoclonal antibodies against Aβ (5). These drugs are primarily capable of alleviating symptoms or delaying disease progression to a limited extent; however, they do not reverse the underlying pathological process. Therefore, it is necessary to explore the pathogenesis and intervention strategies of AD from new perspectives.

Insulin-like growth factor I (IGF-I) is a single-chain polypeptide hormone structurally similar to insulin. It is primarily synthesized by the liver in response to growth hormone stimulation and exerts its effects through either autocrine or paracrine mechanisms (6). Under normal physiological conditions, IGF-I is a key regulator of body growth and development. It helps maintain muscle mass by promoting longitudinal bone growth, stimulating protein synthesis, and inhibiting protein degradation (7). Moreover, IGF-I exerts protective effects on nervous system development and function, supporting neuronal survival, synaptic plasticity, and cognitive processes (8, 9). Under pathological conditions, reduced circulating IGF-I levels are associated with aging, diabetes, cardiovascular diseases, and frailty syndrome (10–12).

A close relationship exists between IGF-1 and AD. Resistance to or reduction of IGF-1 signaling in the brain may exacerbate Tau protein hyperphosphorylation and Aβ deposition, impair synaptic plasticity, and thereby promote the pathological progression of AD (13, 14). Clinical studies have demonstrated that decreased IGF-1 levels in the periphery and cerebrospinal fluid may serve as potential indicators of cognitive decline and disease progression in AD patients (15). Lower basal circulating IGF-1 levels are also associated with an increased risk of developing AD (16). Therefore, targeting IGF-1 could represent a key therapeutic strategy for AD treatment (17).

Bibliometrics is a discipline that employs mathematical and statistical methods to conduct quantitative analysis of academic literature. By examining data such as publication volume, keyword co-occurrence, citation networks, and collaboration patterns among authors and institutions, it objectively reveals the development trends, research hotspots, and cutting-edge directions in specific fields. Applying this approach to the research domain of IGF-1 and AD helps depict the evolution of the field from initial exploration to current hotspots from a macro-level perspective. It enables researchers to quickly grasp shifts in knowledge foundations and research paradigms, accurately identify current research focuses and potential breakthroughs, thereby promoting deeper and more effective development in this multidisciplinary area.

## 2 Methods

### 2.1 Data search

**Figure 1:**
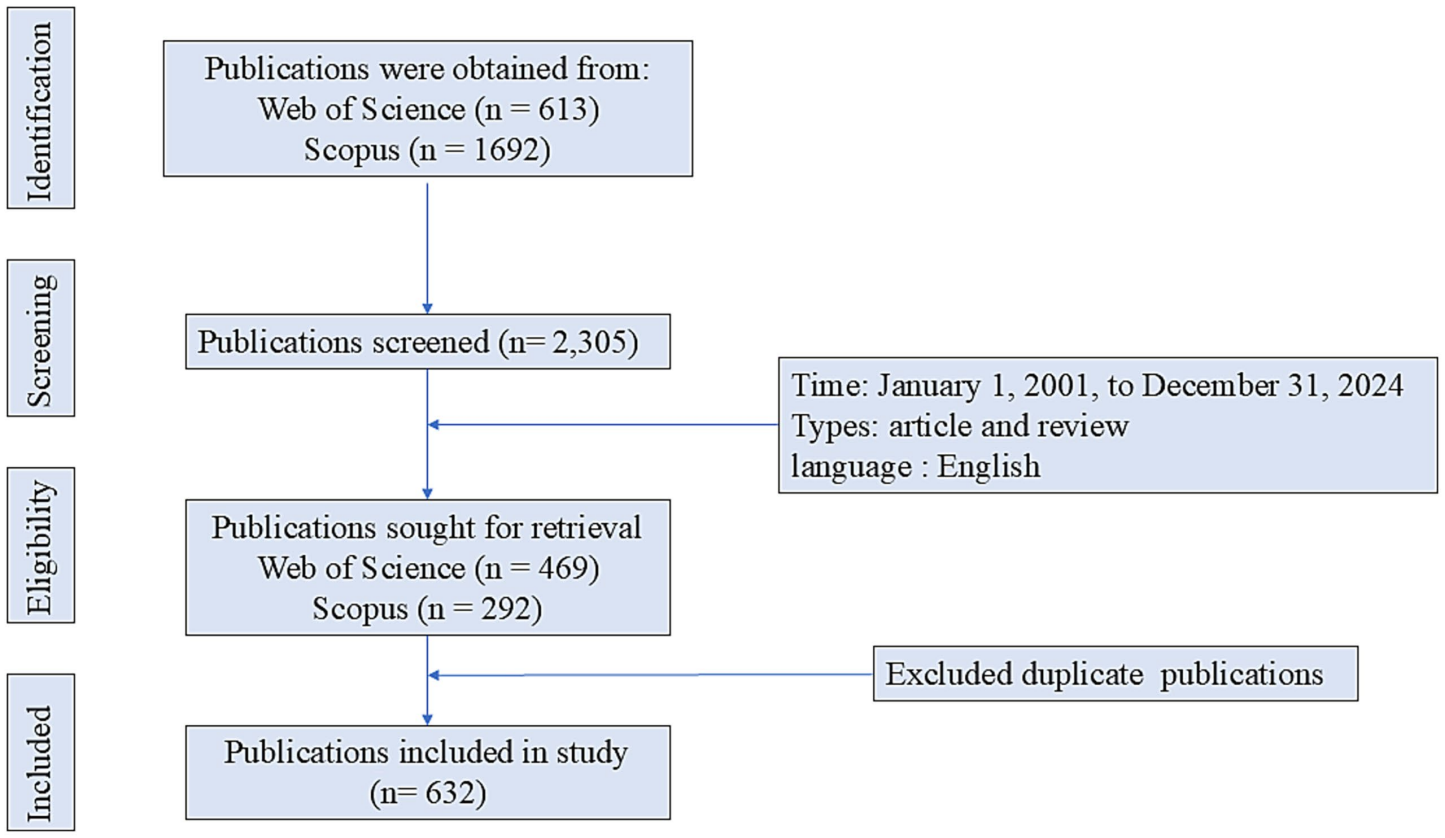
Flowchart of publication search process.

Publications related to IGF-1 and AD were retrieved from the Web of Science (WoS) and Scopus databases. The WoS search formula was: (((((((TS = (Insulin-like Growth Factor 1)) OR TS = (Insulin-like Growth Factor I)) OR TS = (Somatomedin C)) OR TS = (IGFI)) OR TS = (IGF1)) OR TS = (IGF-I)) OR TS = (IGF-1)) AND TS = (Alzheimer’s disease). The Scopus search formula was: (TITLE-ABS-KEY (Insulin-like Growth Factor I) OR TITLE-ABS-KEY (Insulin-like Growth Factor 1) OR TITLE-ABS-KEY (Somatomedin C) OR TITLE-ABS-KEY (IGFI) OR TITLE-ABS-KEY (IGF1) OR TITLE-ABS-KEY (IGF-I) OR TITLE-ABS-KEY (IGF-1) AND TITLE-ABS-KEY (Alzheimer’s disease)). The search was limited to publications published up to December 31, 2024. Only publications in English and of the types “article” or “review” were included. Two researchers independently screened the retrieved records and excluded papers that were irrelevant to the research topic or duplicates. Ultimately, 632 publications on IGF-1 and AD were selected for analysis (Figure 1).

### 2.2 Data analysis

Data analysis and visualization were performed using CiteSpace, VOSviewer, and Bibliometrix, consistent with previously established bibliometric methods (18, 19). CiteSpace focuses on revealing evolutionary trends, emerging frontiers, and critical turning points in a research field, and is employed to analyze developmental trajectories and key pathways of specific topics across different time periods (20). VOSviewer is primarily used to generate and visualize clear and intuitive collaboration and co-occurrence networks; through its density or cluster density views, it effectively highlights core research hotspots and community structures (21). Bibliometrix performs efficient and comprehensive integration, analysis, and visualization of bibliometric data, identifying collaborative networks and disciplinary hotspots, thereby significantly enhancing the depth and efficiency of bibliometric analysis (22).

## 3 Results

### 3.1 Publication trends

**Figure 2:**
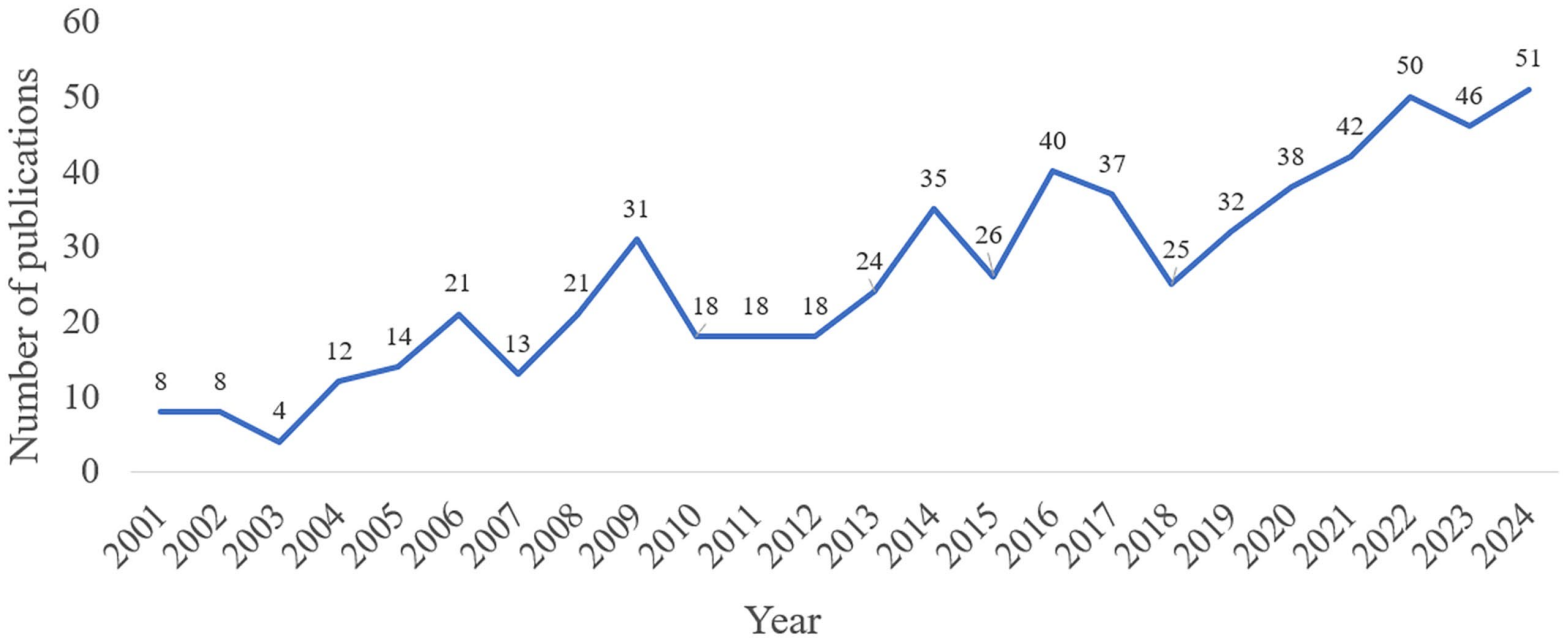
Annual number of publications on IGF-1 and AD.

From 2001 to 2024, the annual number of publications on IGF-1 and AD showed an overall increasing trend (Figure 2). During the initial period (2001–2003), publication output was relatively low, with no more than 10 articles per year, indicating that research in this field was still in its nascent stage. Throughout the fluctuating growth phase (2004–2020), the number of publications increased with some variations, reflecting growing interest in this topic. In the rapid development phase (2021–2024), although there was a slight decline in publications in 2023, the long-term trend still indicated sustained growth and enhanced research activity in this area.

### 3.2 Country

**Figure 3:**
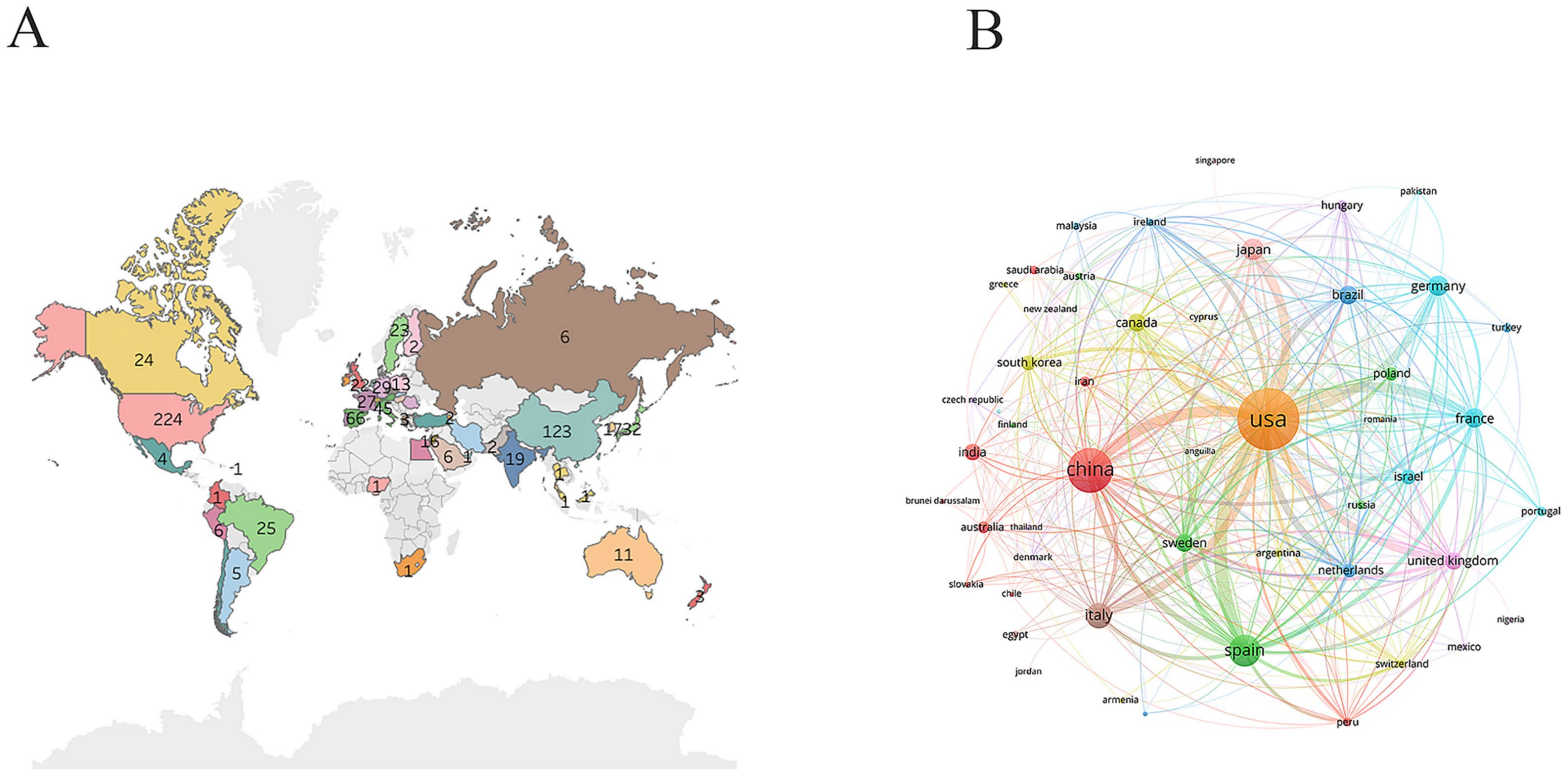
Country analysis. **(A)** Geographic distribution of publications. The marked numbers represent the number of publications from each country. **(B)** International collaborative network. Nodes represent countries, and the lines represent the collaboration between countries. The larger the nodes, the greater the number of publications. The thicker the connections, the closer the collaborative relationship.

The geographic distribution of publications revealed that research was concentrated primarily in North America, Asia, and Europe, with very few studies originating from African countries (Figure 3A). The United States (224 publications) and China (123 publications) were the two most productive countries, significantly outpacing others and underscoring their dominant roles in both research volume and resource investment in this field (Figure 3B). Spain, Italy, and Japan formed a second tier, each producing between 30 and 70 publications. Not only did the United States lead in publication numbers, but it also had the highest total citation count (19,137 times), far exceeding those of other countries, which indicated the considerable academic impact of its research output (Table 1). Italy (45 publications; 3,494 citations) and France (27 publications; 1,811 citations) had relatively high average citations per article, suggesting high research quality. Although China ranked second in total publications, its total citation count (3,227) was comparatively low, indicating potential for improvement in average influence per paper. The United States (total link strength: 1,227) and Spain (total link strength: 558) possessed the most robust collaboration networks, highlighting their central roles in international cooperation and strong collaborative capacity.

**Table 1:** The top 10 countries in the research related to IGF-1 and AD.

Rank	Country	Number of publications	Citations	Total link strength
1	USA	224	19,137	1,227
2	China	123	3,227	443
3	Spain	66	2,610	558
4	Italy	45	3,494	267
5	Japan	32	1,213	296
6	Germany	29	1,173	231
7	France	27	1,811	344
8	Brazil	25	445	144
9	Canada	24	844	217
10	Sweden	23	838	226

### 3.3 Institutions

**Figure 4:**
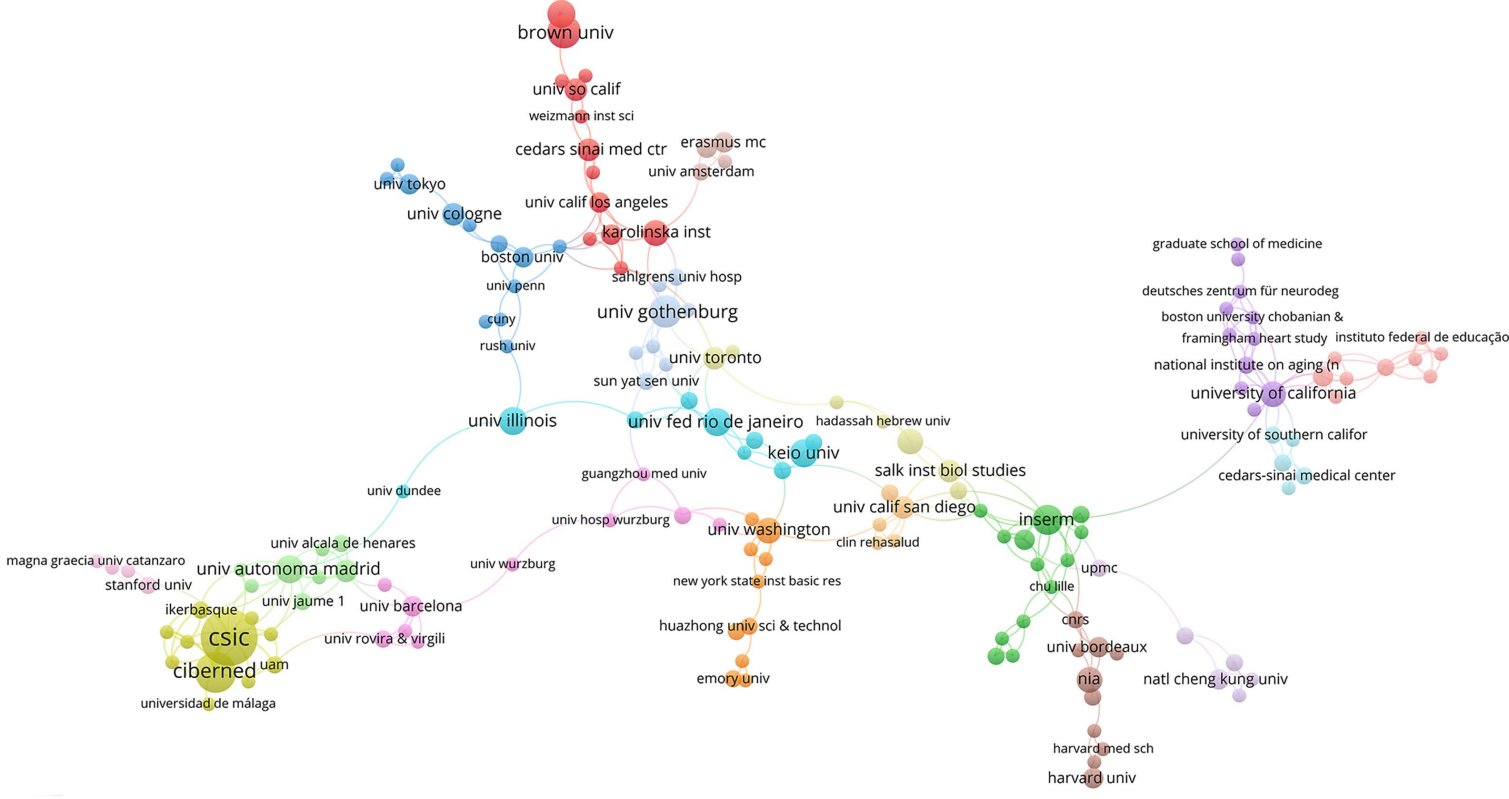
Institutional collaborative network. The size of the nodes represents the number of institutional publications.

Research institutions involved in IGF-1 and AD research were distributed across North America, Europe, Asia, and South America, reflecting worldwide recognition of IGF-1 as a potential therapeutic target for AD. The extent of international collaboration among institutions varied considerably, ranging from highly internationalized partnerships to relatively independent research groups (Figure 4). The Consejo Superior de Investigaciones Científicas (CSIC) led with 26 publications, followed by the Centro de Investigación Biomédica en Red sobre Enfermedades Neurodegenerativas (CIBERNED) (14 publications) and Brown University (10 publications) (Table 2). Spain, relying on an organized research network, has become the world’s most productive research center. The United States had multiple research teams capable of producing highly influential findings. Collectively, these institutions and countries are steering the global research direction regarding IGF-1 and AD.

**Table 2:** The top 10 institutions in the research related to IGF-1 and AD.

Rank	Institution	Documents	Citations	Average number of citations
1	CSIC	26	1,124	43.23
2	CIBERNED	14	261	18.64
3	Brown University	10	1,961	196.10
4	University of Gothenburg	9	282	31.33
5	Inserm	8	618	77.25
6	Rhode Island Hospital	7	2,235	319.29
6	Keio University	7	436	62.29
6	Autonomous University of Madrid	7	280	40.00
6	Federal University of Rio de Janeiro	7	346	49.43
6	University of Illinois	7	244	34.86
6	University of Wisconsin	7	634	90.57

### 3.4 Journals

**Figure 5:**
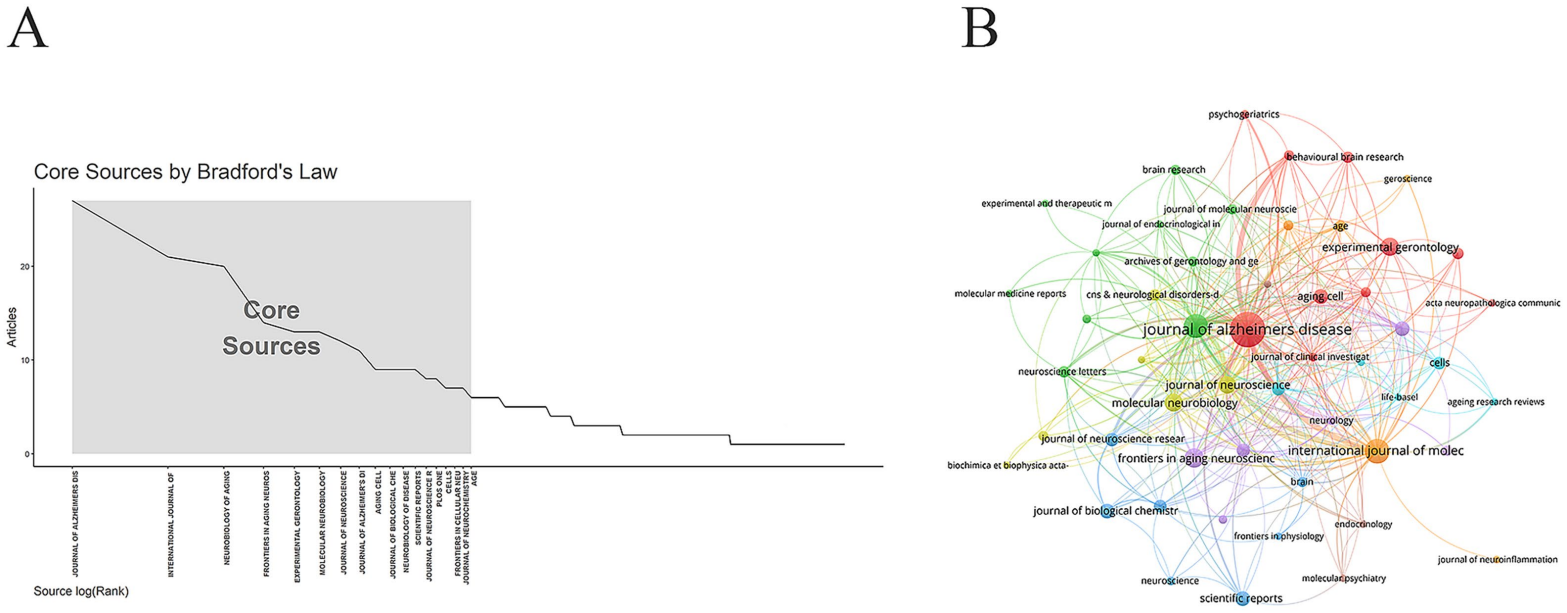
Journal analysis. **(A)** Core journal. **(B)** Journal network. The size of the nodes represents the number of publications in the journals.

According to Bradford’s Law, 17 core journals were identified (Figure 5A), which were highly concentrated in several closely related fields such as neuroscience, aging biology, molecular mechanisms, and disease applications. These journals collectively form a core knowledge cluster focused on brain aging and neurodegenerative diseases. They investigated AD mechanisms extensively—from molecular and cellular levels to systemic perspectives—providing a central academic platform and knowledge foundation for research on the role of IGF-1 in neuroprotection, metabolic regulation, and disease intervention. The journal with the highest number of publications was *Journal of Alzheimer’s Disease* (38 papers), followed by *International Journal of Molecular Sciences* (21 papers) and *Neurobiology of Aging* (20 papers) (Figure 5B and Table 3).

**Table 3:** The top 10 journals in the research related to IGF-1 and AD.

Rank	Source	Documents	Citations	Average number of citations	IF	JCR
1	Journal of Alzheimers Disease	38	2,810	73.95	3.1	Q2
2	International Journal of Molecular Sciences	21	963	45.86	4.9	Q1
3	Neurobiology of Aging	20	1,820	91.00	3.5	Q2
4	Frontiers in Aging Neuroscience	14	363	25.93	4.5	Q1
5	Experimental Gerontology	13	476	36.62	4.3	Q1
5	Molecular Neurobiology	13	703	54.08	4.3	Q1
7	Journal of Neuroscience	12	2,159	179.92	4	Q2
8	Aging Cell	9	637	70.78	7.1	Q1
8	Journal of Biological Chemistry	9	469	52.11	3.9	Q2
8	Neurobiology of Disease	9	222	24.67	5.6	Q1
8	Scientific Reports	9	256	28.44	3.9	Q1

### 3.5 Authors

**Figure 6:**
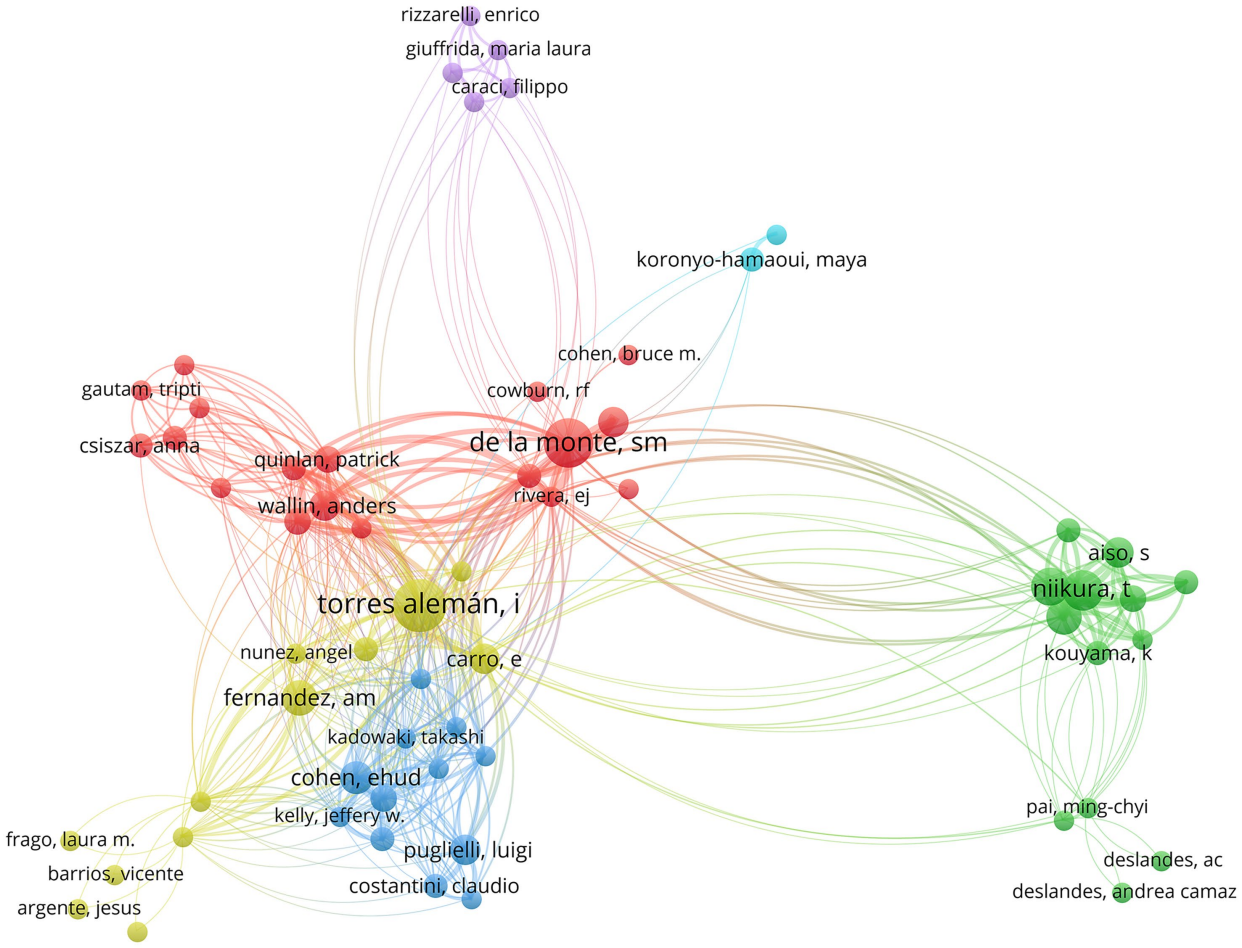
Author collaboration network. The nodes represent the authors, while the lines indicate the collaborative relationships among the authors. The larger the node, the greater the number of publications it represents. The thicker the connection, the closer the collaborative relationship.

Authors with high publication output are generally core contributors and key opinion leaders in the field. Their work often defines research paradigms and guides disciplinary development. High-output authors typically maintain stable core research teams and international collaborations, which serve as a central driving force for the advancement of the field. Nodes of different colors represented different research groups, and the thickness of the connecting lines indicated the degree of collaboration (Figure 6). The most prolific author was Dr. Ignacio Torres-Aleman (16 papers), a renowned neuroscientist from Spain. His research focused primarily on the protective and reparative effects of IGF-1 on neurons, as well as its role in neurodegenerative diseases and brain aging. His team made foundational contributions to understanding how the IGF-1 signaling pathway mediates neurotrophic and anti-inflammatory effects in the brain. Dr. Suzanne M. de la Monte (15 papers) and Dr. Takako Niikura (10 papers) were also among the core authors with substantial publication records in this field (Table 4). Dr. Takako Niikura was one of the key researchers who directly linked the IGF-1 signaling pathway to the pathogenesis of AD, particularly in relation to Aβ toxicity and neuronal apoptosis. The “Type 3 diabetes” hypothesis proposed by Dr. Suzanne M. de la Monte represented a dominant theoretical framework in this area.

**Table 4:** The top 10 authors in the research related to IGF-1 and AD.

Rank	Author	Number of publications	Works	Country	Institution
1	Dr. Ignacio Torres-Aleman	16	(17, 34–48)	Spain	CSIC
2	Dr. Suzanne M de la Monte	15	(49–62)	USA	Brown University
3	Dr. Takako Niikura	10	(63–70)	Japan	Keio University
4	Dr. Yasuhiro Hashimoto	9	(63–68, 70)	Japan	Keio University
5	Dr. Inohiro Nishimoto	8	(63–70)	Japan	Keio University
5	Dr. Ana M. Fernandez	8	(39, 42, 45, 46, 71, 72)	Spain	CSIC
7	Dr. Ehud Cohen	7	(73–78)	Israel	Hebrew University of Jerusalem
8	Dr. Eva Carro	6	(34, 35, 37, 79–81)	Spain	CSIC
8	Dr. Luigi Puglielli	6	(82–87)	USA	University of Wisconsin–Madison
8	Dr. Ming Tong	6	(52–54, 57, 61, 62)	USA	Brown University
8	Dr. Wallin Anders	6	(88–93)	Sweden	University of Gothenburg

### 3.6 Keywords

**Figure 7:**
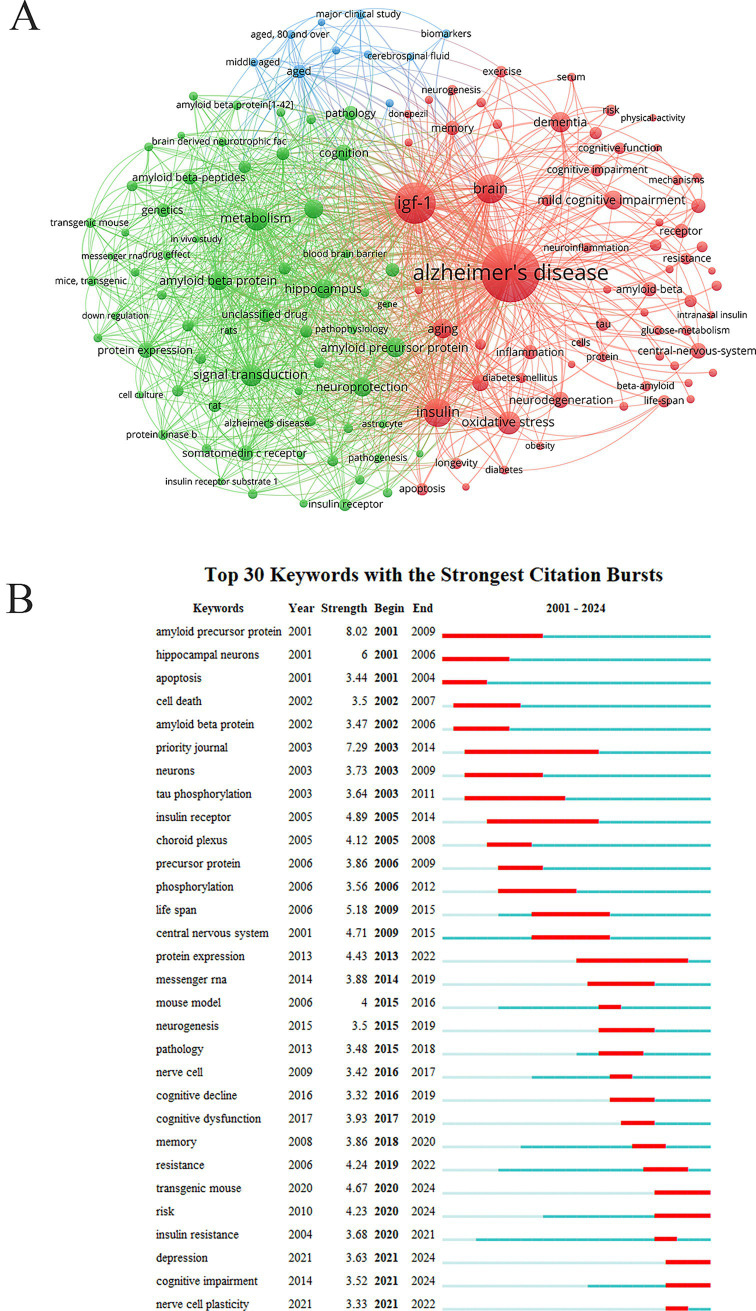
Keywords analysis. **(A)** Keywords network. The size of the nodes represents the frequency of the keywords. **(B)** Keywords with the strongest citation bursts. The red line represents the duration of the outbreak.

High-frequency keywords serve to identify core research hotspots and stable themes. High-frequency keywords in this domain include AD (484 times), IGF-1 (261 times), brain (138 times), insulin (132 times), metabolism (94 times), oxidative stress (92 times), signal transduction (81 times), dementia (72 times), aging (71 times), amyloid beta protein (67imes) neuroprotection (66 times) (Figure 7A). Emergent keywords are those whose frequency increases sharply within a short period, acting as indicators of emerging and rapidly evolving research frontiers. Analysis of temporal trends in research topics revealed that the research paradigms concerning IGF-1 and AD have undergone considerable evolution: initially focusing on basic cellular and molecular mechanisms, later shifting toward animal models and pathological validation, and more recently emphasizing clinical symptoms and comorbidities (Figure 7B). This progression reflected a translational medicine trend, moving from basic research to clinical applications. Initial stage and exploration of basic mechanisms (2001–2008): keywords included amyloid precursor protein, amyloid β-protein, Tau phosphorylation, apoptosis, cell death, and hippocampal neurons. Research during this period centered on the two classic pathological hypotheses of AD: the Aβ hypothesis and the Tau protein hypothesis. Investigations provided fundamental molecular evidence supporting the therapeutic potential of IGF-1. Mechanism expansion and proof-of-concept stage (2009–2016): keywords included lifespan, central nervous system, insulin receptor, phosphorylation, mouse model, and neurogenesis. Researchers began employing mouse models to examine the effects of IGF-1 at the whole-animal level, considering broader outcomes such as lifespan. This stage also involved an in-depth exploration of the interplay between IGF-1 and insulin signaling pathways, thereby strengthening the association between AD and the concept of “type 3 diabetes.” Concurrently, growing interest in neurogenesis highlighted that IGF-1 not only protects existing neurons but also promotes the generation of new ones, suggesting novel therapeutic approaches. Clinical translation and frontier exploration stage (2017–2024): keywords included cognitive decline, cognitive dysfunction, memory, cognitive impairment, resistance, insulin resistance, risk, depression, and neural plasticity. This most recent trend underscored a shift toward clinically relevant outcomes such as cognitive function, comorbid conditions like depression, and deeper metabolic investigations. Emerging keywords such as transgenic mouse, risk, depression, and cognitive impairment represent current and future research directions.

## 4 Discussion

### 4.1 General information

A total of 632 publications were included in the study. The annual publications related to IGF-1 and AD exhibited an overall upward trend. Research was concentrated in North America, Asia, and Europe. The United States holds a dominant position in terms of output, influence, and international influence. The Consejo Superior de Investigaciones Científicas was the most active institution. *Journal of Alzheimer’s Disease* was the journal with the highest number of publications. Dr. Ignacio Torres-Aleman was the most prolific author.

### 4.2 Hotspots and frontiers

The high-frequency keywords “AD” and “IGF-1” occupy the top two positions with an absolute advantage, clearly defining the scope of this research: a focus on IGF-1 as a key molecule and an exploration of its role in AD. “Brain” and “insulin” emphasize the spatial context (central nervous system) and a core theoretical link (the insulin signaling pathway), respectively. The frequent appearance of “insulin” directly points to the “brain insulin resistance” hypothesis and the concept of “AD as type 3 diabetes.” The pioneering work of de la Monte and Wands (23) characterized AD as type 3 diabetes, positing that deficient insulin/IGF-1 signaling in the brain is a key driver of AD pathology, thereby linking metabolic dysfunction with neurodegeneration. IGF-1 resistance and insulin resistance in the AD brain share a common core signaling defect: inhibition of the IRS-1/PI3K/Akt pathway (13). AD-related toxic factors, such as Aβ oligomers and inflammatory cytokines, can induce abnormal serine phosphorylation of IRS-1, concurrently impairing both IGF-1 and insulin signaling (24). Elevated insulin levels compete with IGF-1 for its binding proteins and receptors and downregulate IGF-1 receptor expression in the brain, thereby reducing IGF-1 bioavailability and signaling strength (25). Weakened IGF-1 signaling directly compromises neuronal glucose metabolism and increases susceptibility to energy crisis. Metabolic dysregulation further gives rise to two specific lines of in-depth inquiry. First, oxidative stress has been identified as a key target through which IGF-1 exerts neuroprotective effects. In AD, Aβ deposition and mitochondrial dysfunction lead to excessive reactive oxygen species (ROS) generation and resultant oxidative stress (26). This oxidative stress further damages neurons, exacerbates Aβ and Tau toxicity, and promotes neuroinflammation, forming a vicious cycle. IGF-1 promotes mitochondrial biogenesis, enhances mitochondrial function, and stabilizes mitochondrial membrane potential, thereby reducing mitochondrial ROS leakage and improving neuronal resilience to energy deficit and oxidative stress (27). Second, in-depth analysis of signal transduction has become a major focus of mechanistic research, particularly regarding the PI3K/Akt/GSK-3β and Ras/MAPK pathways downstream of IGF-1R (23). These pathways regulate not only cell survival and energy metabolism but also the phosphorylation of amyloid precursor protein and Tau, influencing Aβ production and Tau hyperphosphorylation. Thus, IGF-1 signaling is closely linked to the core molecular pathology of AD. Ultimately, neuroprotection represents the fundamental goal and translational endpoint of this entire research field. All mechanistic explorations—whether concerning metabolic regulation, antioxidant effects, or signal transduction—converge on the central questions of whether and how IGF-1 can achieve neuroprotection, thereby delaying the progression of AD.

Risk, depression, and cognitive impairment have garnered substantial attention in recent years and may represent emerging frontiers in AD research. Contemporary AD research has shifted from late-stage treatment toward early intervention and risk prediction. Brain IGF-1 resistance or abnormal peripheral IGF-1 levels are considered important risk factors and early events in AD pathogenesis (28, 29). Determining whether IGF-1 levels or related genetic variations can serve as biomarkers for predicting AD risk may facilitate early identification and intervention in high-risk populations. Depression is no longer viewed merely as a reactive psychological consequence of AD; rather, it is recognized as an independent risk factor and prodromal symptom. Before overt memory decline occurs, behavioral changes such as depression and apathy may reflect early manifestations of AD-related brain network pathology (30). IGF-1 can cross the blood–brain barrier and plays a crucial role in learning and memory. It supports cognitive function through various mechanisms, including promoting synaptic plasticity, neurogenesis, dendritic spine growth, and Aβ clearance (30, 31). Impairment of the IGF-1 signaling pathway directly contributes to cognitive decline. The ultimate objective of therapeutic strategies targeting IGF-1 is to evaluate their efficacy in delaying or improving the progression of cognitive impairment.

### 4.3 Clinical research

Currently, clinical observations and pharmacological interventions targeting IGF-1 for the treatment of AD remain relatively limited. A study was conducted to assess the effects of an aerobic exercise training program on brain-derived neurotrophic factor (BDNF), vascular endothelial growth factor (VEGF), and plasma IGF-1 levels, as well as on cognitive function, in patients with AD (Brazilian Clinical Trials Registry, RBR-5q4m7t) (32). The results showed that a 12-week aerobic training program did not significantly improve cognitive function in AD patients or alter the levels of circulating neurotrophic factors. Another 12-month clinical trial (NCT: 00074529) evaluated whether MK-677 (ibutamoren mesylate), a potent IGF-1 secretagogue, could slow symptom progression in AD patients (33). The results indicated that IGF-1 levels were lower in AD patients at baseline. Although MK-677 significantly increased IGF-1 levels, it failed to slow the clinical progression of AD.

### 4.4 Challenge and opportunity

In the field of IGF-1 and AD, current opportunities are as follows: First, basic research has revealed the crucial regulatory role of the IGF-1 signaling pathway in neuronal survival, synaptic plasticity, and Aβ clearance, offering potential targets for developing novel neuroprotective or disease-modifying therapies. Second, advances in precision medicine and biomarkers (e.g., blood or cerebrospinal fluid IGF-1 levels, alone or in combination with neuroimaging) are expected to position the IGF-1 system as a useful auxiliary tool for the early diagnosis, staging, or therapeutic monitoring of AD.

At present, the research also faces some challenges. A major technical hurdle is enabling large-molecule peptide hormones such as IGF-1 to efficiently and stably cross the blood–brain barrier and act precisely on target brain regions, while minimizing systemic side effects such as hypoglycemia and edema. Translating promising results from animal models into clinical benefits for human patients remains a persistent and formidable challenge in the drug development for neurodegenerative diseases. This underscores the necessity for future clinical trials to feature more refined designs and to rely on validated biomarkers for patient selection and outcome evaluation.

## 5 Conclusion

Research on IGF-1 and AD has continued to grow. Studies in this field have formed a tightly interconnected network, centered on the AD pathological core—IGF-1-related molecular mechanisms—downstream signaling pathways. The research focus is shifting from superficial correlations to investigations into underlying mechanisms and potential therapeutic targets. Depression and cognitive impairment are likely to become promising frontiers for future research.

## Data Availability

The original contributions presented in the study are included in the article/supplementary material, further inquiries can be directed to the corresponding authors.
